# Advertising Crime

**Published:** 1900-10

**Authors:** 


### ADVERTISING CRIME.

THE article published in the August JOURNAL, concerning the printing by daily newspapers of advertisements of quacks, charlatans, fakirs and abortionists, has attracted general attention on the part of the medical journals, and in some clean newspapers the evil has been commented on and the crusade endorsed.

The Mississippi *Medical Record*, published at Vicksburg, Miss., has begun a similar crusade in the South and attacks the evil with courage and a most refreshing energy.

The editors of the *Record* do not content themselves with merely calling attention to the curse of "medical advertising" in the daily newspaper, but they go beyond that and openly attack the professional abortionist, boldly and fearlessly and denounce the curse in unequivocal terms. In an editorial on the subject, the *Record* says:

Referring to an editorial, The Criminal Abortionist, in the August issue of the *Record*, the question has been asked the editors if it is not possible to have the license of the criminal abortionist revoked?

There is no law on the statute books of this state authorising the revocation of the license of a doctor for any crime whatever. That such a law ought to exist we have no doubt. In a crime of this kind it would doubtless be much easier to convict where the penalty would be the revoking of license than where it is death or imprisonment for life. While an action revoking the license of a doctor for the act of criminal abortion would not bar an indictment and trial before the courts, it would in a measure restrict the crime of abortion by licensed physicians. . . . .

This method if adopted would be a good move in the right direction; but if the advertising privileges of the fakir and the abortionist were curtailed, they would sooner or later be driven out of business. As it is now there is a public standing invitation in almost every issue of every newspaper published in Buffalo, pointing the way to unfortunate or foolish women, to the slaughter houses where they may be aborted. One advertiser, in fact, has gone so far as to open up a shady "private hospital" in a building with an unsavory reputation, where he performs his operations, and from which he may carry out his dead in daylight. And this man's advertisements are carried into every home in the City of Buffalo where the English afternoon newspapers are read. Thus far, not one Buffalo newspaper has announced the intention of exercising editorial supervision over its advertising columns.

How differently the management of Frank Leslie's Monthly looks at the matter is shown in this editorial statement recently made:

> Advertisements for the cure of the opium, morphine and liquor habit; rupture; teaching of hypnotism, mesmerism and mind cure, and others of the objectionable medical class will be refused admission to our pages.
>
> Any advertisement of any description which we believe is deceptive in its claims, or offensive to good taste, will be declined. It is our purpose to admit only announcements which are thoroughly reliable, and of a clean, desirable character; and to exercise a strict editorial censorship over the advertising pages.

There is still hope that Buffalo newspaper proprietors will reach the conclusion that a lot of honest decency is more desirable than a few questionable dollars.

---

THE *Philadelphia Medical Journal* in its issue of September 1, 1900, prints the following commentary on code-worship that deserves reading by every physician in the State of New York, not to say every one in the United States. It is a short, incisive paragraph, that groups into a few sentences all there is left of the so-called code controversy in this state. But we need not elucidate; let Dr. Gould speak for himself. These are his words:

> Idolatry in code-worship is a tendency that seems more and more prevalent. One of the most honored and honorable of American physicians recently protested that consultation with sectarians and stealing patients was more prevalent with some code-worshipers (citing the cases of some principal officers of great medical organisations) than with practitioners excluded from the ranks of the faithful. There is, of course, a usefulness in the antiquated code, but we should not forget that zealots are prone to worship the idol rather than the spirit it represents. Idolatry may be as bad as or worse than atheism. Certainly there is no reason for pride on the part of the idolaters, who disobey the spirit of ethics in their practice, and who excuse the most outrageous professional scoundrelism if it happens to be exhibited by the code-worshipers. One should set his own household right before lecturing his neighbors. It is a standing disgrace that all reputable regular physicians cannot join our national medical organisation because of a silly war of words. All who love our profession more than their "logic," cliqueism, or vanity, will surely try to find a common meeting-ground and a method of uniting upon essentials.

---

WHEELING, W. Va., has an epidemic of rabies on its hands, and a city ordinance has been passed and is being enforced requiring that all dogs be confined or muzzled. Thus far, there have been two deaths from rabies. Judging from Buffalo's experience Wheeling has a long, hard fight ahead.

---

THE New York Board of Health has decided to erect a one-story building near the Willard Parker Hospital, to be used as a bacteriological laboratory solely for the purpose of studying the plague. This is a case where a politician is not out of place as President of the Board of Health, for the idea was suggested by Col. Michael C. Murphy and carried out by him.

